# Dental Undergraduates and Interns' Awareness, Attitudes, and Perception of Radiological Protection

**DOI:** 10.1155/2022/5812627

**Published:** 2022-05-09

**Authors:** Elfatih Abuelhia, Ali Alghamdi, Abdulrahman Tajaldeen, Osama Mabrouk, Adel Bakheet, Haney Alsaleem, Wejdan Alaraik, Amir MSmar, Faisal Quwaihes, Khaled Alshahrani, Yahya Hlosh, Salem Alghamdi, Rowa Aljondi

**Affiliations:** ^1^Department of Radiological Sciences, College of Applied Medical Science, Imam Abdulrahman Bin Faisal University, Dammam, Saudi Arabia; ^2^Department of Research Statistical Support, Deanship of Scientific Research, Imam Abdulrahman Bin Faisal University, Dammam, Saudi Arabia; ^3^Department of Applied Radiologic Technology, College of Applied Medical Sciences, University of Jeddah, Jeddah, Saudi Arabia

## Abstract

Medical ionizing radiation is widely used in hospitals, in particular dental clinics, and in medical research to facilitate the diagnosis and treatment of patients. The awareness, attitude, and perception of ionizing radiation exposure among dental undergraduate students and interns in radiological investigations and dental care clinics were investigated. A cross-sectional study was conducted; 17 questions were designed online using the software “QuestionPro,” which was licensed to the University of Imam Abdulrahman Bin Faisal. Participants included senior medical dental students from Imam Abdulrahman Bin Faisal University in their third to fifth years, as well as interns from King Fahad University Hospital and private dental care clinics. A total of 855 participants viewed, 360 started the questionnaire, and 258 (72%) completed it online. Overall, knowledge was lacking; 32% of respondents incorrectly believed that magnetic resonance imaging and ultrasound used ionizing radiation, while 38% were unsure. Dental X-rays were deemed harmful by 40% (*n* = 104) of respondents. According to 33% (*n* = 85) of participants, there is no radiation scatter during an X-ray or CT scan, while 30% (*n* = 76) are unsure. Respondents (44%; *n* = 104) were unaware of the radiation dose from a chest radiograph and (45%; *n* = 116) overestimated the radiation dose. The effects of ionizing radiation on healthy tissue are known to more than half of the participants (54%). According to 39% of respondents, digital radiography exposes them to less radiation than traditional radiography. In terms of radiation protection and hazard, 46% said personal monitoring badges should be always worn and 58% (*n* = 150) said lead aprons should be used on a regular basis. 63% of the subjects had received radiation protection education, such as formal lectures, tutorials, or workshops, while 37% (*n* = 95) had not. 53% of the respondents were not aware of the international recommendations from the International Commission on Radiological Protection. When asked if they would follow radiation protection protocols if they opened a private dental clinical practice in the future, 50% (*n* = 129) said they would.

## 1. Introduction

Ionizing radiation is produced when an unstable atom's nucleus decays and begins to cause ionizing particles to be released. Ionizing radiations, such as X-rays, contain enough energy required to extract an electron from an atom, resulting in the formation of free radicals that are chemically unstable and very reactive in the process [1]. When these particles encounter human tissue, they will cause burns and cancer if the levels are sufficiently high. Ionizing radiation is harmful to most living tissues and can be a lifetime risk, causing cancers such as leukemia and genetic damage. The development of X-ray imaging around the late 1800s was one of the most significant advances in medical science [2]. Ionizing radiation emitted by diagnostic imaging sources such as X-rays and computed tomography is not without danger. However, the advantages of using X-rays for diagnostic purposes in both medicine and dentistry are enormous [3]. Over the last two decades, the number of studies involving ionizing radiation has increased dramatically. Radiation is thought to have dose-dependent negative effects on the human body, raising the risk of cancer. When ionizing radiation interacts with human living tissue at the atomic level, biological effects occur [4]. These biological effects are categorized into two types: deterministic effects occur when the magnitude of the response is proportionate to the absorbed dose and below a certain dose, the response is not noticeable. And nondeterministic effects, in which the likelihood of the change occurring, rather than its severity, is considered, depends on the absorbed dose, and does not have dose thresholds. Both the patients and the operational personnel are in a high-risk situation [5]. Radiation dangers are detrimental, and it becomes dangerous when professionals are negligent or ignorant. Although the exposure is considered modest, dental professionals and patients must be subjected to the least amount of radiation feasible due to a lack of understanding and measures to avoid the detrimental consequences of the radiation. A dental radiograph is ordered when the benefit of disease detection outweighs the risk of X-ray radiation damage [6]. Many experts have reported a paucity of information and guidance from dental specialists regarding the X-ray radiation dose requested for radiological imaging in Saudi Arabia. For example, Assiri and his colleagues' work demonstrated that dental referring doctors' knowledge was adequate, but protective measures should be improved specifically for dental X-ray hazards [7]. Almohaimede et al. conducted a similar study and reported that radiation risks were well understood; however, radiation safety precautions should be stressed more among general practitioners in governmental and private sectors [8]. Ionizing radiation doses were not well understood or widely known in imaging radiology among young clinicians and senior medical students within the study fields [9]. In the detection of several dental problems, CT, especially its derivatives 3D CT and ortho cubic super-high-resolution CT, and the clinically applicable artificial intelligence system recently become the method of choice [10, 11]. Radiation from CT has a significant danger of harming some categories of patients; notably, youngsters are more vulnerable to ionizing radiation harm than others, emphasizing the importance of ensuring optimal radiation use [12]. A review study was conducted by Reda et al. on the possible application of magnetic resonance imaging (MRI) in dentistry, radiation-free diagnostic exam; they highlight the potential of MRI for diagnosis in dental clinical practice, without the risk of biological damage from continuous ionizing radiation exposure [13]. The study's goal is to assess dental undergraduate students' and interns' knowledge, awareness, attitudes, and perception of ionizing radiation exposure in radiological imaging at King Fahad University Hospitals, Imam Abdulrahman University, and private dental clinics in Saudi Arabia's Eastern Province.

## 2. Materials and Methods

A cross-sectional study was conducted, and a questionnaire was created online utilizing software “QuestionPro,” which was licensed to the University of Imam Abdulrahman Bin Faisal. Participants included senior medical dental students from Imam Abdulrahman Bin Faisal University in their third to fifth years, as well as interns/junior doctors from King Fahad University Hospital and private dental care clinics. The questionnaire was divided into three parts. The first part is about the participant's demographics, their education, as well as a working knowledge of ionizing radiation and the international regulations recommended by ICRP. The second part examined the participants knowledge about the equipment used in radiological investigations and whether they produce ionizing or nonionizing radiation. In this section, participants were asked if they had any education on radiation protection. Moreover, participants were asked if the ultrasound and MRI machines produce ionizing radiation. In the final part, the participants were assessed on their knowledge of the estimated radiation dose during dental x-rays, the biological effects, and the hazards of ionizing radiation. Finally, the participants were asked about their personal protection and if they have private clinical practice in the future they would take care of radiation protection protocols.

### 2.1. Statistical Analysis

The data were subsequently processed and analyzed using the Statistical Package for the Social Sciences for Windows (SPSS version 25). The Pearson chi-square test was done to evaluate the statistical significance. For individual and multiresponse analysis in differential statistics, the comparison among groups was evaluated by the ANOVA test. The statistical significance level was chosen at *P* ≤ 0.05. The correct response of participants toward radiation hazards and protection was graded into low (<50%), average (51%–75%), and good (>75%).

## 3. Results

**Figure 1:**
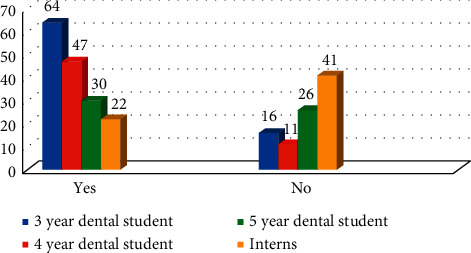
Education and knowledge of participants on radiation protection (*χ*^2^ = 49.64 with *P* ≤ 0.05).

**Figure 2:**
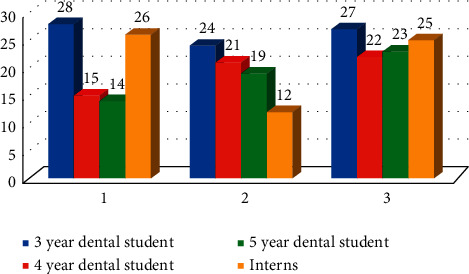
Awareness of participants if ultrasound and MRI involve ionizing radiation (*χ*^2^ = 7.54 with *P* > 0.274).

A total of 855 participants viewed online, 360 started the questionnaire, and 258 (72%) were fully completed. 147 were male (57.65%) and 108 were female (42.35%). 30.86% were third year dental students (*n* = 79), 22.66% of fourth year dental students (*n* = 58), 21.88% of final year dental students (*n* = 56), and 24.61% of interns (*n* = 63). When participants asked if they had ever done radiation protection studies, 63.42% (*n* = 163) responded in the affirmative and 36.58% did not. Difference between the responses in all four groups was statistically significant (*χ*^2^ = 49.64 with *P* ≤ 0.05) (Figure 1). Several participants (32.42% (*n* = 83)) wrongly stated that ionizing radiation was used in ultrasonography and MRI, while 37.89% (*n* = 97) did not know; difference between the responses in all groups was statistically nonsignificant (*χ*^2^ = 7.54 with *P* > 0.05) (Figure 2).

**Figure 3:**
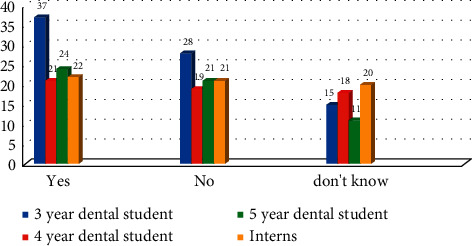
Response of participants if dental X-ray is harmful (*χ*^2^ = 5.60 with *P* > 0.469).

Among the participants, 40.47% (*n* = 104) thought that dental X-rays are harmful and 24.9% (*n* = 64) do not know. Difference between the responses in all four groups was statistically nonsignificant (*χ*^2^ = 5.60 with *P* > 0.05) (Figure 3).

**Figure 4:**
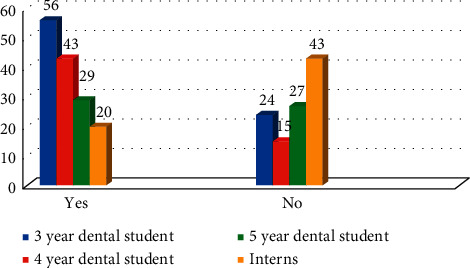
Knowledge of participants toward the benefits of using collimators and filters in dental radiography (*χ*^2^ = 29.55 with *P* ≤ 0.01).

Medical imaging technicians who employ ionizing radiation are expected to understand how radiation interacts with matter and the process of radiation scattered within the medium. When the participants asked about whether during X-ray or CT scan, the X-ray radiation can be scattered from the walls, 33.07% (*n* = 85) and 29.57% (*n* = 76) stated no and do not know, respectively. Difference between the responses in all four groups was statistically nonsignificant (*χ*^2^ = 5.94 with *P* > 0.05). To reduce the exposure of the patient to ionizing radiation during radiological investigations, the participants were assessed if they knew the benefits of using collimators and filters in dental radiography. 42.41% (*n* = 109) incorrectly stated that no benefits of using collimators or filters, while 57.59% (*n* = 148) said yes. Difference between the responses in all four groups was statistically significant (*χ*^2^ = 29.55 with *P* ≤ 0.01) (Figure 4). This was the highest rate of correct answers, with reference to the imaging questions.

The response to the question about the estimated radiation dose in mSv of a chest X-ray. Only 10.2% (*n* = 26) of the participants correctly estimated the radiation dose, which is approximately 0.02 mSv, while 45.48% (*n* = 106) were incorrectly stated and 44.31% (*n* = 113) did not know. The difference between the responses in all four groups was statistically nonsignificant (*χ*^2^ = 6.876 with *P* > 0.05) (Table 1).

**Figure 5:**
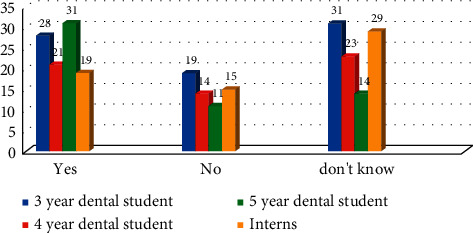
Awareness of participants toward digital radiography compared to conventional radiography in exposure difference (*χ*^2^ = 9.479 with *P* > 0.148).

The subject's knowledge of the patient's exposure to the radiation dose during X-ray was determined to have the least reliable answers. According to 39% of respondents, digital radiography exposes them to less radiation than traditional radiography. Difference between the responses in all four groups was statistically nonsignificant (*χ*^2^ = 9.479 with *P* > 0.05) (Figure 5).

**Figure 6:**
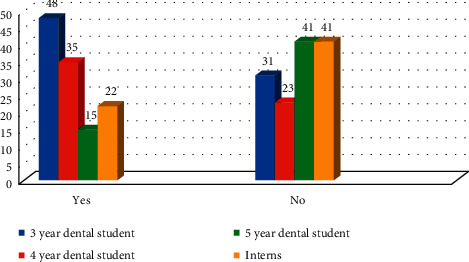
Awareness of participants toward the international recommendations from the ICRP (*χ*^2^ = 23.03 with *P* ≤ 0.01).

The participants were asked if they are aware of the international recommendations from the International Commission on Radiological Protection (ICRP) regarding the ionizing radiation principles. The difference in responses across all four groups was statistically significant (*χ*^2^ = 23.03 with *P* ≤ 0.01) (Figure 6).

The purpose of dental radiography is to gather important diagnostic information while minimizing radiation exposure to the patient and dental staff. The dental unit's operator must be at least six feet away from the main beam or behind a protective barrier (operator position him/herself at an angle ranging from 90° to 135° from the center ray). If a protective barrier is employed, it must feature a viewing glass so that the operator can see the patient.  Table 2 provides the responses of participants' awareness of the knowledge of the dental unit operator position.

Exposure to low levels of radiation encountered in dental radiography procedures does not cause an immediate health effect but is a minor contributor to our overall cancer risk in the log time effect.

**Figure 7:**
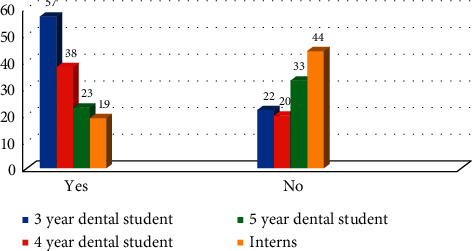
Knowledge of participants toward ionizing radiation effects on healthy tissue (*χ*^2^ = 31.69 with *P* ≤ 0.01).

Are you aware of ionizing radiation effects on healthy tissues? Difference between the responses in all four groups was statistically highly significant (*χ*^2^ = 31.69 with *P* ≤ 0.01) (Figure 7).

**Figure 8:**
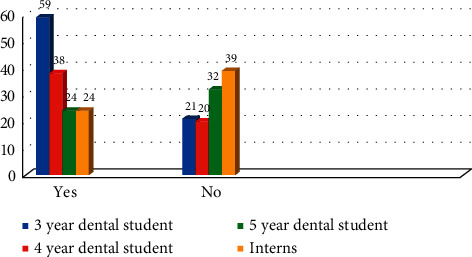
Awareness of participants toward radiation hazard signs (*χ*^2^ = 24.52 with *P* ≤ 0.01).

When the participants asked about their knowledge and awareness of the ionizing radiation hazard signs, total of 146 (56.4%) responded correctly. The difference in the responses in all four groups was statistically significant (*χ*^2^ = 24.52 with *P* ≤ 0.01) (Figure 8).

**Figure 9:**
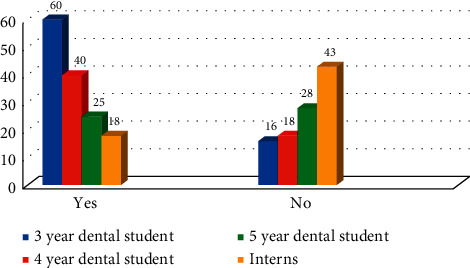
Response of participants toward the use of lead apron in regular basis (*χ*^2^ = 39.34 with *P* ≤ 0.01).

The participants asked about the routine use of lead aprons on a regular basis. The students from 3^rd^ year, 4^th^ year, 5^th^ year, and interns replied yes in proportions of 78.9%, 69%, 47.2%, and 29.5%, respectively. Difference between the responses in all four groups was statistically highly significant (*χ*^2^ = 39.34 with *P* ≤ 0.01) (Figure 9).

## 4. Discussion

Assessing the impact of low radiation exposure on health has become a research priority. Exposure to ionizing radiation occurs in a variety of occupational categories, including dental procedures used in medical imaging, which results in low-level radiation exposure. Although the impact of dental X-rays on patients and practitioners is considered minor, it should not be underestimated. Dental clinicians, in general, require adequate training and updates on radiation hazards and the protective guidelines that must be followed. This cross-sectional questionnaire has the potential to provide dental clinicians with invaluable information during their early education period. Saudi researchers are concerned about the use and risk of low-level ionizing radiation in dental clinicians in healthcare or private hospitals and they are looking for an alternative radiation-free diagnostic possibility in dental clinical [14–17]. The participants in this study were asked more than 9 questions about radiation hazards and protection. 40.5% of participants agreed that dental X-rays are harmful, while 60% either disagreed or did not know. These findings were inconsistent and lower than those published by Assiri H et al., 55.06%, Basheer et al., 63.5%, and Shah et al., 77.5% [7, 15, 18]. When patients undergo dental procedures, other very sensitive organs, such as the thyroid, bone marrow, and brain, which are not the intended target, may receive small amounts of radiation and may cause biological effects. The participants were asked about radiation scatter and the importance of using collimators and filters in accordance with radiation protection guidelines. 58% of those polled agreed that collimators and filters should be used to protect other organs such as the thyroid. Our findings are consistent with those of Basheer et al., whose participants stated that the thyroid should be protected during dental X-rays. Other medical modalities, such as intraoral and panoramic radiographs, are used in dental practice daily. The participants were asked about the radiation scattered from the wall. Only 37% stated yes. In similar studies conducted by Arnout et al., and Basheer et al., 69.7% and 54.6% of undergraduate students answered yes, respectively [14, 15]. Individual patients may also benefit from CBCT. The participants were asked how much absorbed dose they received during a computed tomography chest X-ray. Respondents (44%; *n* = 104) did not know the radiation dose from a chest X-ray and (45%; *n* = 116) overestimated the radiation dose. The participants were asked to estimate how much absorbed dose they received during a computed tomography chest X-ray. The participants were asked about radiation exposure during digital radiography procedures. 39% of respondents are aware that digital radiography exposes people to less radiation than traditional radiography. In a similar study conducted by Eman et al., 68.0% of participants responded that they would follow a radiation protection policy in their future clinical practice. Half of the participants in this study said will follow the radiation protection regulations and safety in their future clinical practice. Ionizing radiation could cause biological effects if it is used without knowledge of the principles and guidelines established locally and internationally. In this work, participants were asked about their understanding of the recommendations and guidelines of the ICRP. Despite the fact that 63% of the subjects had received radiation protection education, such as formal lectures, tutorials, or workshops, and 37% (*n* = 95) had not, 53% of the respondents were unaware of the ICRP's international recommendations. To increase the low confidence in radiation knowledge and to help in conveying the hazards to patients, further education is required [19]. Certain limitations in this study must be considered in future studies to accurately measure the awareness, attitudes, and perceptions of dental undergraduates and interns. Study limitations, such as a lack of adequate sample size, respondents' honesty, and limitations in study design or methodology, make it difficult to reach more accurate conclusion, furthermore, even gender distribution as well as participants from both public and private hospitals.

## 5. Conclusion

Knowledge of ionizing radiation in radiological imaging is crucial for avoiding serious biological impacts as well as the negative consequences associated with overexposure. Doctors are required by law to obey ionizing radiation restrictions, both globally and locally, as well as basic health and safety norms. Although 54% participants understand the effects of ionizing radiation on healthy tissue, the general level of understanding was low; 32% of respondents wrongly assumed that MRI and ultrasound employ ionizing radiation, while 38% did not know. In terms of radiation protection and hazards, knowledge was higher, with 58% believing that lead aprons should be used on a regular basis. The results emphasize the significance of incorporating the subject into the curriculum of medical dentist students. Education is still the most important source of radiation protection knowledge. To enhance the findings, we suggested that radiation protection and safety classes should be included as part of on-the-job training and that a radiological examination request should be preevaluated, and a local radiology instructional website accessible via the intranet to all clinicians, including up-to-date information on ionizing radiation and patient care, should be created.

## Figures and Tables

**Table 1:** Awareness of participants of approximate radiation dose, in mSv of chest X-ray.

Approximate radiation dose in mSv	3^rd^ year dental student (%)	4^th^ year dental student (%)	5^th^ year dental student (%)	Interns (%)	Total (%)
20	15 (18.8)	7 (12.1)	11 (19.6)	11 (17.5)	44 (17.1)
2	9 (11.3)	8 (13.8)	6 (10.7)	11 (17.5)	34 (13.2)
0.2	12 (15)	10 (17.2)	5 (8.9)	11 (17.5)	38 (14.8)
0.02	9 (11.3)	5 (8.6)	8 (14.3)	4 (6.3)	26 (10.1)
Do not know	35 (43.8)	28 (48.3)	26 (46.4)	26 (41.3)	115 (44.7)
Total (100%)	80	58	56	63	257

**Table 2:** Dental unit operator must stand at least six feet from the useful beam or behind a protective barrier.

Operator position in a dental unit	3^rd^ year dental student (%)	4^th^ year dental student (%)	5^th^ year dental student (%)	Interns (%)	Total (%)
Yes	27 (34.2)	27 (46.6)	33 (60)	27 (42.9)	114 (44.7)
No	12 (15.2)	9 (15.5)	9 (16.4)	12 (19.0)	42 (16.5)
Do not know	40 (50.6)	22 (37.9)	13 (23.6)	24 (38.1)	99 (38.8)
Total (100%)	79	58	55	63	255

## Data Availability

The data used to support the findings of the study are available from the corresponding author upon request.
